# STEM crisis teaching: Curriculum design with e‐learning tools

**DOI:** 10.1096/fba.2020-00049

**Published:** 2020-08-16

**Authors:** Sonya E. Van Nuland, Elissa Hall, Natalie R. Langley

**Affiliations:** ^1^ Department of Cell Biology and Anatomy Louisiana State University Health Sciences Center New Orleans LA USA; ^2^ Office of Applied Scholarship and Education Science Mayo Clinic College of Medicine and Science Mayo Clinic Rochester MN USA; ^3^ Department of Laboratory Medicine and Pathology Division of Anatomic Pathology Mayo Clinic Arizona Scottsdale AZ USA

**Keywords:** computer‐assisted instruction, COVID‐19, curriculum, distance education

## Abstract

The COVID‐19 pandemic and subsequent social distancing protocols have accelerated the shift to online teaching across the globe. In Science, Technology, Engineering, and Mathematics (STEM) programs this means a shift from face‐to‐face laboratory instruction to self‐directed learning with e‐learning tools. Unfortunately, selecting and integrating an e‐learning tool into a curriculum can be daunting. This article highlights key questions and practical suggestions instructors should consider in choosing the most effective option for their course and learners.

## 1 INTRODUCTION

On March 11, 2020 the World Health Organization (WHO) declared the novel coronavirus (COVID‐19) outbreak a global pandemic^.^ ^1^ In the weeks that followed, educational institutions worldwide closed brick and mortar classrooms and made a rapid transformation to online learning and remote content delivery.^2^, ^3^, ^4^, ^5^, ^6^ Over the past three decades online education has grown considerably and now is central to the long‐term strategies of many higher educational institutions. Competing responsibilities of learners have, in part, driven the development of flexible learning opportunities, enabling learners to learn synchronously or asynchronously from a distance. The COVID‐19 pandemic changed online education from an alternative to a necessity.^7^, ^8^ In biomedical sciences education, this meant a shift from laboratory teaching to self‐directed learning using e‐learning tools.^3^, ^9^ Now, more than ever, e‐learning tools have become a valuable asset in delivering content and learning experiences.^10^ However, selecting an e‐learning tool can be frustrating if faculty are not versed in criteria for critically evaluating the number and variety of e‐learning tools.^11^ This article highlights key principles to consider when selecting and integrating an e‐learning tool into a curriculum, which may be helpful—if not essential—as educators transition to distance learning in an online environment.

## 2 WHAT ARE E‐LEARNING TOOLS AND APPLICATIONS?

For the purposes of this article, e‐learning tools are computer applications that mediate the learner's interaction with educational content through an electronic interface to facilitate knowledge construction.^12^, ^13^ The use of e‐learning tools in the biomedical sciences prior to the COVID‐19 pandemic has been well‐documented in undergraduate Science, Technology, Engineering, and Mathematics (STEM) programs (e.g., zoology, physics, chemistry, engineering, nursing, medicine). STEM educators may choose from many options, and the choices are multiplying rapidly as demand increases with the switch to remote teaching and learning on virtual platforms. Many education institutions have created their own e‐learning solutions or invested in commercial options over the past two decades. Some commercial examples include LabsLand^©^ and Complete Anatomy^©^, while institutional creations include 360Anatomy (Western University, London, Canada; 360anatomy.uwo.ca) and SecondLook (University of Michigan; https://secondlook.med.umich.edu).^14^ Many e‐learning platforms (e.g., VoiceThread, Turning Point^®^, Poll Everywhere) also integrate with learning management systems (e.g., Blackboard^®^, Desire2Learn, Moodle^®^, etc). The realities of educating in a socially distanced environment are likely to increase pressure to incorporate e‐learning tools into STEM curricula.

## 3 NAVIGATING THE SEA OF CHOICES

Introducing a new e‐learning tool can be daunting when selection, implementation, and integration must happen swiftly. We outline three broad questions, based on common considerations and pitfalls (Table 1), to help educators select and integrate an e‐learning tool that complements the curriculum and addresses learners’ needs.

**Table 1:** Selecting and integrating an e‐learning tool: common pitfalls, complications, and oversights

Consideration	Common Pitfalls
Skills/content	Unclear expectations of the skills and content you want learners to learn with the e‐learning tool Selecting a tool that does not deliver knowledge or facilitate skill development expected of the learners Using an unnecessarily complex e‐learning tool to teach simple concepts
Usability	Selecting a tool that is difficult for faculty and learners to use Assuming learners will be intuitive users of the e‐learning tool Assuming commercial e‐learning tools have been developed and evaluated for learning effectiveness Selecting a tool with a limited life span or inadequate support Choosing a tool that does not satisfy the budgetary or security requirements of your institution Selecting a tool that does not integrate with various operating systems or learning management systems Choosing a tool that cannot be customized to your needs
Curricular alignment	Selecting a tool that does not align with the course learning objectives, assessments, or instructional methods Choosing a tool that does not facilitate the cognitive levels specified in the learning objectives or promote understanding and mastery of the content Selecting a tool that does not align with how you assess knowledge, skills, and competencies Underestimating the effort and time required to integrate the tool constructively into the learning experience

1. What skills or content do you want learners to learn with the e‐learning tool?

The dynamic nature of various STEM disciplines (physics, chemistry, engineering) may make them well‐suited to using e‐learning tools to enhance learning through simulation. E‐learning tools that facilitate experiential learning support learners’ understanding of physical models and processes and make interactions at the particle level less abstract.^15^, ^16^ E‐learning tools provide opportunity for learners conceptualize structures that cannot be seen with the naked eye, such as atoms or cellular structures, and visualize complex processes such as protein synthesis or the operation of efficient machines, processes, and systems. Research suggests e‐learning tools with two‐ or three‐dimensional animations and interactive components benefit learners who study highly dynamic subjects such as mechanical systems, computer algorithms, or geological and astronomical phenomena.^16^, ^17^, ^18^, ^19^, ^20^

However, e‐learning tool effectiveness seems to be tied to the nature of the content. In anatomy, static content (e.g., neurovascular and osteological structures) may not require highly interactive e‐learning tools with animations. Instead simpler e‐learning tools that present like a textbook may depict the relevant morphology and anatomic relationships adequately and effectively. For example, take two commercial anatomical e‐learning tools that have dissimilar interface designs:^21^

1. A.D.A.M. Interactive Anatomy (Ebix, Atlanta, GA): a two‐dimensional tool that presents static anatomical images in a textbook‐like fashion. This e‐learning tool only allows users to view anatomical structures from “key views” (i.e., anterior and posterior) and has relatively low interactivity (nine features), meaning that students interact with the program using a limited number of features.
2. Netter's 3D Interactive Anatomy (Elsevier, Philadelphia, PA): a three‐dimensional appearance and provides a high level of user interactivity (20 features). In this program users are also able to rotate a structure along any axis point, enabling unlimited viewing angles.

Research involving these tools suggests that the simple e‐learning tool (A.D.A.M. Interactive Anatomy) is as effective as the more complicated e‐learning tool (Netter's 3D Interactive Anatomy).^21^ Furthermore, results demonstrated that Netter's 3D Interactive Anatomy significantly disadvantaged learners with lower spatial ability, while A.D.A.M. Interactive Anatomy did not academically disadvantage any learners.^21^ In fact, other studies also indicate anatomical science e‐learning tools that allow learners to rotate and manipulate unfamiliar objects on a virtual platform may hinder learning processes for learners with lower spatial abilities.^21^, ^22^, ^23^, ^24^, ^25^, ^26^, ^27^, ^28^, ^29^, ^30^, ^31^, ^32^ However, it is important to stress that dynamic concepts such as muscle actions and physiological processes (e.g., blood circulation through the heart) may be ideally suited for highly interactive e‐learning tools, rather than simpler options. Analyzing the concepts you teach and how you plan to assess learners on their knowledge of these concepts will help you to decide whether a complex, highly interactive e‐learning tool versus a more simple, static tool is suitable for learners’ needs.

2. Can you navigate the e‐learning tool efficiently, effectively, and satisfactorily?

Usability is the extent to which a product facilitates goals with effectiveness, efficiency, and satisfaction.^33^, ^34^ Successful e‐learning tool interaction requires learners to navigate the platform, orient themselves to how the information is presented, and then filter and synthesize the subject matter.^13^, ^35^ An e‐learning tool that requires a learner to spend considerable time learning how to navigate the program may compromise their understanding of the educational content.^36^, ^37^, ^38^, ^39^ Poor usability may frustrate learners and discourage adoption.^40^, ^41^ Similarly, poor usability may aggravate educators and lead to clumsy integration of the tool into the curriculum, affecting the overall learning experience and reducing the likelihood that learners use the tool. To mitigate negative outcomes, educators should demonstrate how to integrate the e‐learning tool into their learning template at the beginning of the course. This may take the form of a short screen recording providing learners with a basic understanding of how to navigate the program and/or an in‐class demonstration of the tool (either in person or virtually with screen sharing). These demonstrations reduce frustration and allow learners more time to use the e‐learning tool to aid, plan, and coordinate their learning.

Usability is a complex construct that involves more than the learner implications discussed above. Other usability considerations should be assessed from a course‐design perspective including:

1. What is the “lifetime” of the e‐learning tool? Will it work for future needs or other curricular needs? Tools with limited life span or applications may face premature sunsetting and require considerable re‐design of course assessments and delivery methods.
2. Do you require an e‐learning tool that is readily implemented, or one that offers creative control to customize it to your needs? If the latter, what is the learning curve, and do you have adequate technical support at your institution or from the e‐learning tool provider?
3. Does the tool satisfy your institution's security requirements? For example, some institutions have strict guidelines about the use of usernames and passwords to access web‐based content. Others require educators to go through an extensive approval process to ensure the platform is secure. You should consult with your Information Technology department early in the selection process to speed implementation.
4. Is the e‐learning tool available across various operating platforms (e.g., Windows, Mac, web‐based, desktop, mobile)? This consideration is especially important, as learners vary in their preference for operating systems and platforms. Prescribing a single technology platform may disadvantage learners who are accustomed to a different platform.
5. Is there a cost associated with the e‐learning tool? If yes, then inquire about licensing options and educator discounts as well as consult the appropriate decision makers at your institution.

3. Can the e‐learning tool be aligned to your curriculum meaningfully and constructively?

Curricular alignment is best achieved with backward design, an educational planning approach that uses outcomes to design curriculum units, performance assessments, and classroom instruction.^42^ The same approach should be used to align an e‐learning tool with a curriculum, since this tool will deliver content and, potentially, assessments. By beginning with the end in mind, educators ensure that the e‐learning tool is aligned seamlessly and constructively with all course components (i.e., learning objectives, instructional methods, and assessments.^43^ This requires considerable time and effort on the front end, but the pay‐off and time saved on the back end are invaluable.

The backward design process involves three stages:

1. Identify desired results (i.e., learning objectives and outcomes: what the learners should know, understand, and be able to do).
2. Determine how you will assess the desired results have been achieved (e.g., formative and summative assessments).
3. Design activities that ensure learners achieve these results (e.g., lesson plans, learning events, and teaching methods that provide the knowledge and skills needed to achieve the outcomes).

Questions you should ask to ensure the e‐learning tool facilitates the alignment of objectives, assessments, and instructional methods include:

1. What type of learning experience do I want to deliver (e.g., in‐person lectures and/or activities, blended learning, or distance learning)? This helps you decide if the tool should be a central part of the content delivery and assessment of learner learning or an optional/supplementary resource. For example, when designing an anatomy curriculum, distance learning through a technology platform may require an e‐learning tool to deliver content conventionally taught with cadaveric dissection. In this instance, an e‐learning anatomy application that learners consult as an atlas during dissection transitions from a supplemental reference resource to an essential means of presenting content and assessing knowledge.
2. Does the e‐learning tool support the instructional method (e.g., lecture, team‐based learning, problem‐based learning, flipped classroom)? This will help select key features offered by the tool, such as adaptive learning, built‐in quizzes, or the ability to create and deliver content asynchronously. For example, as educators flip the classroom, built‐in quizzing features integrated with asynchronously delivered didactic content is significant to facilitate self‐assessment of knowledge acquisition in preparation for the synchronous course session.^44^ To facilitate open discussion, application, and inquiry during a synchronous session, educators will seek e‐learning tools which support small and large group formats and provide a real time, collaborative workspace.^44^
3. What features are required to deliver content and/or assessments in your course (audio recordings, video recordings, integration with the learning management system (LMS), mobile friendly applications, etc.)? If the content is highly visual in nature, video recordings are a desirable feature. If you are replacing in‐person laboratory experiences, the tool may need to offer a means to engage learners in simulated activities or experiments. If you would like learners to access it via the LMS and/or their mobile phones, the tool needs to integrate with these platforms. When replacing in‐person laboratory experiences with simulated activities delivered with an e‐learning tool, it is critical to revise and align assessments accordingly. For example, if laboratory dissection is replaced with a three‐dimensional virtual anatomy platform with interactive models, learner knowledge of anatomic structure should be evaluated on the platform, not on images of cadaveric dissections.
4. What level of interactivity will be required for the learners to accomplish learning outcomes, and does the e‐learning tool provide the appropriate level and options? Consider whether the tool is intended to be a supplementary learning resource or reference, or if it serves as a central component of content delivery, self‐directed learning, and/or assessment of learning.
5. Does the e‐learning tool facilitate the cognitive domain specified in the learning objectives (as ascribed by Bloom's taxonomy)? Bloom's taxonomy defines and distinguishes different levels of cognition using a hierarchical ordering from lower cognitive levels (e.g., memorization) to higher levels (e.g., evaluation and application). The taxonomy provides a framework for specifying expectations, or objectives, of what learners should accomplish as a result of instruction.^45^ Higher levels of cognition such as evaluating and creating may not be achievable with all e‐learning tools, so special consideration should be given to aligning the e‐learning tool capabilities with learning objectives.^46^
6. Would you like to be able to track data and user performance to assess learner engagement and progress? Would you like learners to be able to track data, receive immediate feedback, and assess their own performance? If so, inquire if the tracking features integrate with your LMS and/or assessment dashboard. You may also want to investigate if the application offers adaptive learning—a data‐driven approach that provides customized learning paths to address the unique needs of each learner. Adaptive learning provides learners with immediate, customized feedback to advance their learning.

**FIGURE 1:**
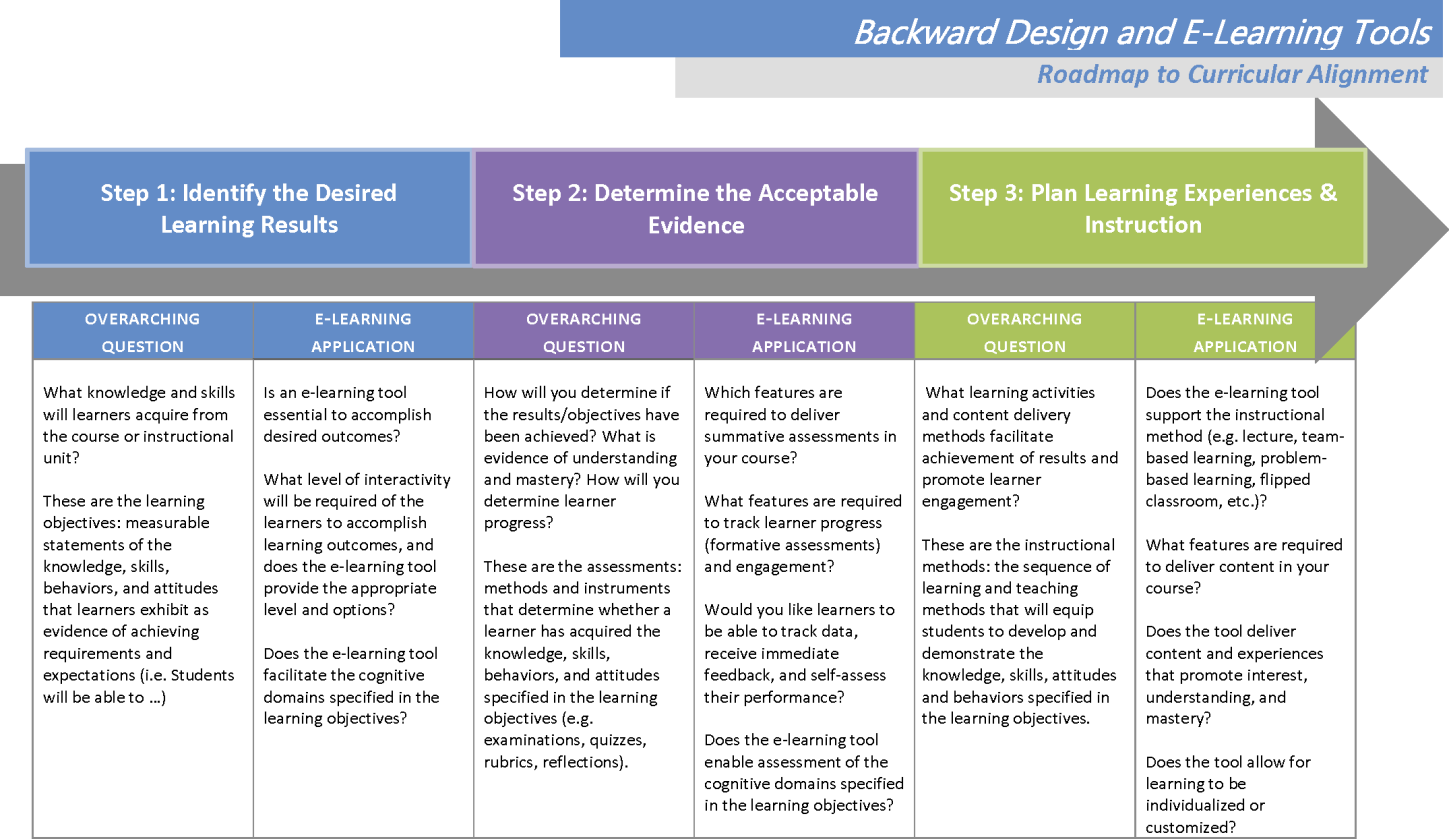
Using backward design to align an e‐learning tool constructively into a curriculum

The questions provided here are not exhaustive; however, they will help align an e‐learning tool with your curriculum and the needs of the learners, educators, and institution. The backward design process should be used to thoughtfully incorporate an e‐learning tool into any curriculum. This process is critical to achieving desired learning outcomes and fostering a positive learning environment. Figure 1 summarizes the backward design process for curricular alignment, with an emphasis on e‐learning tool integration.

## 4 BEWARE OF YOUR ASSUMPTIONS

The authors encourage educators to challenge their assumptions about e‐learning tools.^21^, ^34^ Global commercial entities produce and market e‐learning products and services in a competitive marketplace, and often there is no quality assurance mechanism to protect consumers or learners.^47^, ^48^ As a result, extensive variability exists in the quality and usability of commercial e‐learning tools, with no guarantee that they have been developed and evaluated for learning effectiveness.^49^, ^50^ Similar concerns exist with e‐learning tools developed by educational institutions, as tools may not rest upon sound pedagogical and cognitive principles, but instead on the aesthetics of the technology.^34^

## 5 CLOSING THE LOOP

The necessity of integrating e‐learning tools offers educators and researchers an opportunity to contribute data to inform post‐pandemic educational decisions. Evaluating the learning and efficacy of integrated e‐learning tools is important given the scarcity of reliable evidence and controlled studies in the educational literature.^51^ Education science researchers who contribute to this body of literature will ensure e‐learning tools are effective and reliable, subject to continuous quality improvement, and not a novelty with a limited life span.^44^

## 6 CONCLUSION

The COVID‐19 pandemic has compelled the rapid implementation of online learning to accomplish outcomes and sustain educational programs. STEM faculty are looking to e‐learning tools to facilitate what used to be face‐to‐face laboratory experiences in an online environment. This article highlights key principles and questions educators should consider to select and integrate e‐learning tools into curricula. E‐learning tool integration is complex and rarely a smooth journey; frontline educators who have committed to making innovative changes in their curricula in this time of uncertainty should be commended.

## CONFLICT OF INTEREST

The authors have no conflict of interest to declare.

## AUTHOR CONTRIBUTIONS

S.E. Van Nuland wrote the first draft of the manuscript. S.E. Van Nuland, E. Hall, and N.R. Langley jointly developed the structure and arguments for the paper and contributed to the writing of the manuscript. All the authors reviewed and approved the final manuscript.
